# Evaluation of gene-expression clustering via mutual information distance measure

**DOI:** 10.1186/1471-2105-8-111

**Published:** 2007-03-30

**Authors:** Ido Priness, Oded Maimon, Irad Ben-Gal

**Affiliations:** 1 Department of Industrial Engineering, Tel Aviv University, Israel

## Abstract

**Background:**

The definition of a distance measure plays a key role in the evaluation of different clustering solutions of gene expression profiles. In this empirical study we compare different clustering solutions when using the Mutual Information (MI) measure versus the use of the well known Euclidean distance and Pearson correlation coefficient.

**Results:**

Relying on several public gene expression datasets, we evaluate the homogeneity and separation scores of different clustering solutions. It was found that the use of the MI measure yields a more significant differentiation among erroneous clustering solutions. The proposed measure was also used to analyze the performance of several known clustering algorithms. A comparative study of these algorithms reveals that their "best solutions" are ranked almost oppositely when using different distance measures, despite the found correspondence between these measures when analysing the averaged scores of groups of solutions.

**Conclusion:**

In view of the results, further attention should be paid to the selection of a proper distance measure for analyzing the clustering of gene expression data.

## Background

In recent years, DNA microarray technology has become a vital scientific tool for global analysis of genes and their networks. The new technology allows simultaneous profiling of the expression levels of thousands of genes in a single experiment. At the same time, the successful implementation of microarray technology has required new methods for analyzing such large scale datasets. Clustering is a central analysis method of gene-expressions that has been implemented extensively in various works and applications [1-5]. The primary goal is to cluster together genes or tissues that manifest similar expression patterns [1]. The underlying assumption is that co-expressed genes or tissues with correlated pathways may share common functional tasks and regulatory mechanisms. Similar expression patterns might offer insights into various transcriptional and biological processes [6-8].

Many clustering algorithms depend heavily on 'similarity' or 'distance' measures (although not necessarily a distance function that satisfy all mathematical conditions of a metric) that quantify the degree of association between expression profiles. The definition of the distance measure is a key factor for a successful identification of the relationships between genes and networks [6]. Different similarity measures are likely to result in different clustering, although based on the same expression data.

Despite the crucial influence of the similarity measure upon the clustering results, there are fewer publications on this subject in the bioinformatics literature. Many publications focus on the efforts to optimize and justify the implemented biological processes and the clustering algorithms, while the similarity measures are often selected by default [6,8,16,37-39]. As indicated in [16]: "*Clustering co-expressed genes usually requires the definition of 'distance' or 'similarity' between measured datasets, the most common choices being Pearson correlation or Euclidean distance... it is widely recognized that the choice of the distance may be as crucial as the choice of the clustering algorithm itself (D'haeseleer et al., 2000). However, as pointed out by Brazma and Vilo (2000), the appropriateness of similarity measures has not been systematically explored and these measures are used on an ad-hoc basis*." Many publications use traditional clustering algorithms (e.g., K-means, Self Organizing Maps and Artificial Neural networks) that have roots in conventional data-extensive research fields, such as signal or image processing. In these fields, the similarity measures rely on the unique characteristics of the specific data structure. For example, in signal processing it is commonly assumed that identical codeword vectors are distorted by white noise components during transmission. Under such an assumption, it is reasonable to use a vector-quantizer encoder which is based on the Euclidean distance [9]. We claim that the same reasoning is not necessarily applicable to the analysis of gene expression profiles. Thus, further attention should be paid to the selection of a proper distance measure for analyzing the clustering of gene expression data.

In addition to the Euclidean distance, another widely used measure for analyzing and clustering gene expression data is the Pearson correlation coefficient [1,10-14]. It is used despite its underlying assumption on the linear relationships between genes' expressions. As opposed to these measures, it is well known that *mutual information *(MI) provides a general measurement for dependencies in the data, in particular positive, negative and nonlinear correlations (e.g., [15,16]). This property is important to identify genes that share inputs to which they respond differently [17].

Within the large body of research on gene expression clustering, there are few publications that systematically explore the appropriateness of chosen similarity measures. Herzel and Grosse (1995) [15] analyze the relationships between various correlation functions and the MI. They emphasize that the MI can detect any kind of dependence between patterns. Michaels et al. (1998) [17] present a strategy for the analysis of large-scale quantitative gene expression data from time course experiments. They consider two distance measures: the well established Euclidean distance and a normalized MI. The authors present their approach mainly to demonstrate the essence of the MI measure, and state that further study is required to assure robustness. This paper follows their suggestion and uses known datasets to measure the robustness of clustering solutions based on the Euclidean distance, the Pearson correlation and a normalized MI measure. Steuer et al. (2002) [16] and Daub et al. (2004) [18] investigate the use of MI as a distance measure for gene expression data. They also focus on the comparison between the MI and the Pearson correlation measures.

Most of the above papers, with the exception of Daub et al (2004) [18], mention the similarity between the MI measure and the conventional ones. In particular, Michaels et al. (1998) [17] indicate that the Euclidean distance and the MI measure have a high degree of correspondence. Steuer et al. (2002) [16] conclude that within the investigated dataset there seems to be almost a one-to-one correspondence between the MI and the Pearson correlation measures. A similar observation is supported in this study by finding a high correspondence level in the behaviour (e.g., trends) of average scores that are based on different distance measures. Nevertheless, this study shows that within the analyzed datasets, the MI-based scores better differentiate among clustering solutions of different quality when compared to the other distance measures.

This paper proposes a procedure to evaluate the MI between gene expression patterns. Consequently, by using several public gene expression datasets, it compares the MI measure with respect to both the Euclidean distance and the Pearson correlation. The comparison includes a consistency examination upon clustering solutions of different quality in terms of the number of errors. The clustering is carried out by using normalized homogeneity and separation functions that provide a uniform scale for the examination. The results of the first experiment clearly show that the MI outperforms the conventional measures by yielding a more significant differentiation among clustering solutions. Next, the paper employs the MI measure to evaluate the solutions of four recognized clustering algorithms over a yeast cell-cycle database [19]. This known database has been traditionally used to examine various algorithms and techniques for gene expression analysis [5,20,21]. The results show that the sIB algorithm [32,33], which is originally based on a mutual-information criterion, obtains better MI-based homogeneity and separation scores than those provided by the K-means, the CLICK and the SOM algorithms [5,21]. These results totally change when evaluating the same solutions by the Pearson correlation based homogeneity and separation scores.

The remainder of paper is organized as follows. The Results section describes two experiments: the first experiment compares the robustness of the distance measures and the second experiment evaluates the solutions of known clustering algorithms by both the MI based scores and the Pearson correlation based scores. The Discussion and Conclusion sections follow the Results section. The Methods section addresses the compared distance measures and their implementation to clustering; the assessment of the quality of the clustering solutions, and the compared clustering algorithms.

## Results

### Experiment 1: Robustness of compared distance measures

The underlying idea in this experiment was to evaluate the performance of the three distance measures based on clustering solutions with a known number of clustering errors. Given a dataset with true two-clusters solution, we generated several erroneous solutions having a different number of errors. Clustering errors were generated by transferring samples from their true cluster to the erroneous one. Given a dataset with *N *samples correctly clustered into two groups, the maximum number of errors is generated by misclassifying *N*/2 samples (misclassifying *l *samples is equivalent to misclassifying the remaining *N*-*l *samples). Since the number of different solutions with *l *errors out of *N *samples grows exponentially with the number of errors (up to *l = N*/2), the solutions with more than a single error were generated randomly (without repetition) to obtain 50–60 different solutions depending on the size of the dataset.

In the next stage, the generated clustering solutions were grouped by their quality level, i.e., by the number of errors with respect to the true solution. The average homogeneity and separation scores were calculated for each group based on each of the three similarity measures. Finally, the "robustness" of each similarity measure was defined and evaluated according to the conformation with the following two criteria.

• A monotonic relationship between the obtained scores and the quality of clustering solutions. The smaller is the number of errors in a solution, the better should be its homogeneity and separation scores and vice versa.

• Statistically-significant differentiation between clustering solutions of different quality level. The average homogeneity and separation scores for each similarity measure and for each group were evaluated empirically from the experiments. Accordingly, it is expected that scores of groups of different quality will significantly differ from each other. Such a differentiation assures that the scores of high-quality clustering solutions will not get "mixed up" with the scores of low-quality clustering solutions, hence, our use of the term "robustness".

The first criterion is mainly affected by the trend of the averaged scores of clustering solutions as their quality changes. The second criterion is more rigorous in a sense, and tries to establish a statistically-significant differentiation between groups of clustering solutions based on the scores' mean values and the scores' standard deviations. In fact, a similarity measure that complies with the second criterion guarantees a high power of a statistical test. In our case, this criterion decreases the Type II statistical error, thus the probability to reject a false null hypothesis that two different clustering solutions belong to the same quality group.

#### Datasets

In this part of the experiment we used four public gene-expression datasets that are listed in Table 1. Each dataset contains two types of samples with a clear biological distinction, leading to a 'true' bi-clustering solution. For this reason, we focused here on sample (tissue) clustering rather than gene clustering, whose 'true' solution is less evident. Each of the first three datasets is composed of two types of tumorous samples, originally taken from Su et al. (2002) [22]. These datasets were processed on the Human genome U95 Affymetrix microarrays. The gene pool was obtained by selecting the most up-regulated genes for each sample. We used the data, which is available at [23], as it was used by Monti et al. (2003) [24]. The fourth dataset is composed of healthy and tumorous samples of colon tissue, known as the "Colon cancer dataset" from Alon et al. (1999) [3]. Of the ~6000 genes represented in the experiment, 2000 genes were selected based on the confidence in the measured expression levels. This dataset is available at [25].

**Table 1:** Used datasets for the experiment 1

#	No. of tissues (samples)	No. of genes	Min H.ratio	Min S.ratio
1	28 lung cancer/23 colon^1 ^cancer	1 K	1.47	1.65
2	26 breast cancer/28 lung cancer	1 K	1.42	1.83
3	26 breast cancer/23 colon^1 ^cancer	1 K	1.12	1.32
4	40 colon^2 ^cancer/22 normal colon	2 K	1.02	0.93

List of the used datasets for experiment 1 including their sizes. The last two columns represent the ratio of the Homogeneity (H) and the Separation (S) Z-scores between the MI measure and the best score from among the Pearson correlation and the Euclidean distance. These ratios are calculated based on 10-errors clustering solutions.

#### Experimental results

**Figure 1:**
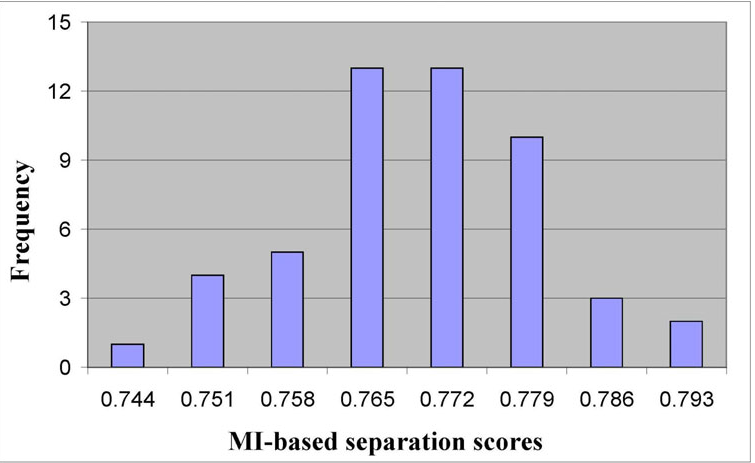
**A normal-shape frequencies of MI-Separation scores**. A normal-shape frequencies of the MI-based separation scores of the single-error group of solutions for dataset 1 that contains 1000 genes from 51 sampled tissues.

The average homogeneity and separation scores for each group were normalized to provide a uniform scale for the comparison of the three similarity measures. The normalization of the average scores were calculated with respect to the mean and the variance values of the group of single-error solutions. Thus, the obtained Z-scores reflect the difference in the average values in terms of the number of standard deviations of the single-error group of solutions. Note that the homogeneity and separation scores of the single-error groups of solutions were approximately normally distributed for almost all the distance measures and all the datasets. For example, in Figure 1 the frequencies of the MI-based separation scores of the single-error group of solutions from dataset 1 are depicted.

**Figure 2:**
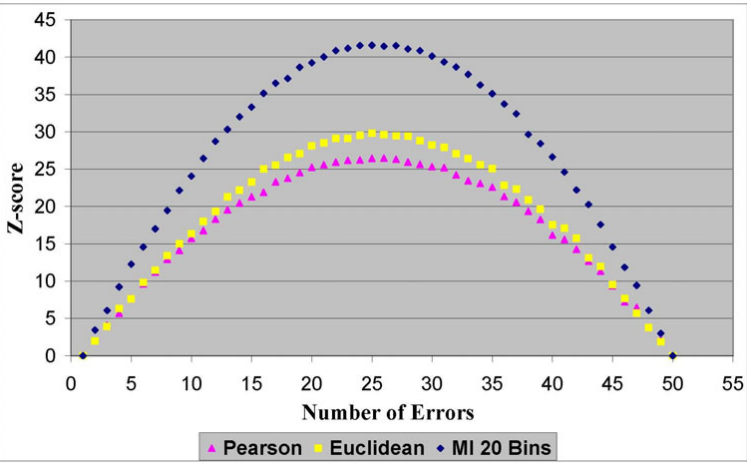
**Homogeneity Z-scores for dataset 1**. Normalized homogeneity Z-scores of clustering solutions with different number of errors based on the Pearson correlation, the Euclidean distance and the MI measures for dataset 1 that contains 1000 genes from 51 sampled tissues.

**Figure 3:**
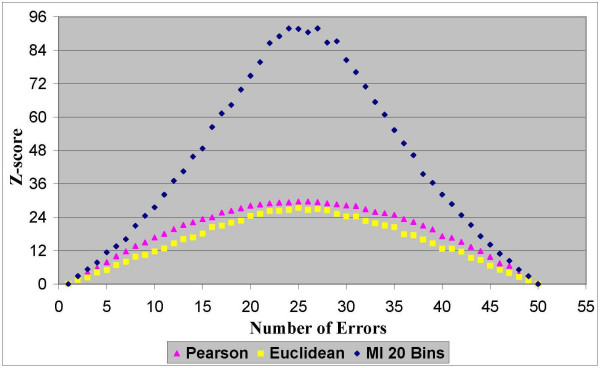
**Separation Z-scores for dataset 1**. Normalized separation Z-scores of clustering solutions with different number of errors based on the Pearson correlation, the Euclidean distance and the MI measures for dataset 1 that contains 1000 genes from 51 sampled tissues.

**Figure 4:**
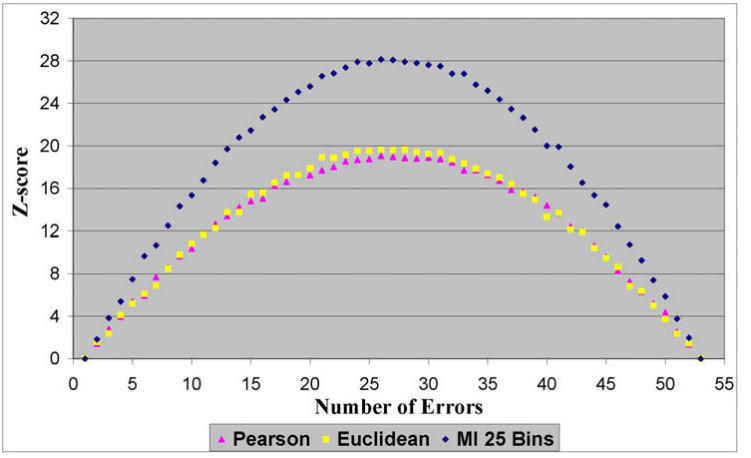
**Homogeneity Z-scores for dataset 2**. Normalized homogeneity Z-scores of clustering solutions with different number of errors based on the Pearson correlation, the Euclidean distance and the MI measures for dataset 2 that contains 1000 genes from 54 sampled tissues.

**Figure 5:**
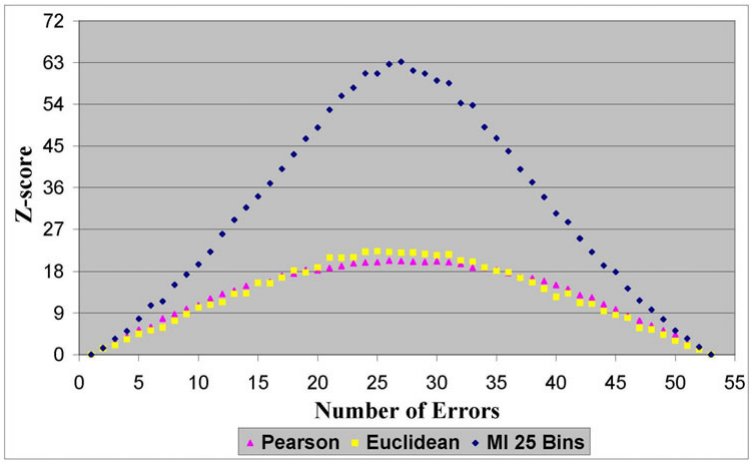
**Separation Z-scores for dataset 2**. Normalized separation Z-scores of clustering solutions with different number of errors based on the Pearson correlation, the Euclidean distance and the MI measures for dataset 2 that contains 1000 genes from 54 sampled tissues.

In the first experimentation stage we found that all three similarity measures fully meet our first criterion of "robustness". Namely, for all the distance measurements and for all the datasets the scores demonstrated a consistent capability to evaluate the quality of the clustering solutions. The results in Figures 2, 3, 4 and 5 clearly show that homogeneity and separation scores worsen as the number of errors increases. Moreover, similar scores were obtained for clustering solutions with an equivalent number of errors, resulting in a symmetric score function with respect to the number of errors.

In terms of the second criterion, the results from all four datasets (two datasets are depicted in Figures 2, 3, 4 and 5 and the other two are depicted in the additional appendix file [56]) clearly show that the MI-based scores outperform the Pearson correlation and the Euclidean distance. The MI-based homogeneity and separation yields a more significant differentiation among clustering solutions of different quality in terms of their errors. For example, in clustering solutions of dataset 1 the average ratio between the MI normalized homogeneity score and the Pearson/Euclidean normalized homogeneity score is 1.5. To give a sense of this ratio in terms of the normal probability distribution which is supported in Figure 1, let us consider the difference between single-error solutions and double-error solutions. In such a case the normalized homogeneity score based on Pearson/Euclidean measures stands on 2 standard deviations (with a corresponding *p *value of 0.023), while the normalized homogeneity score based on the MI measure stands on 3.45 standard deviations (with a corresponding *p *value of 0.0003). We find that for any number of clustering errors higher than one, the obtained MI-based scores are statistically more significant than the Pearson-based or Euclidean-based scores. Therefore, the use of MI-based scores results in a smaller type-II error (or a higher power of test) in comparison to the other distance measures when used to evaluate the quality of a clustering solution. For example, Figures 2 and 3 present respectively the homogeneity and separation scores based on the three compared distance measures for dataset 1. Figures 4 and 5 present the same scores for dataset 2 (figures for the other datasets can be found in the additional appendix file [56]). The fourth (fifth) column in Table 1 presents the minimum ratio between the MI homogeneity (separation) score and the Pearson/Euclidean homogeneity (separation) score for solutions with 10 errors.

Despite the intrinsic tradeoff between the homogeneity and the separation scores, the MI-based scores clearly dominate the other distance measures for all the number of errors. Similar results were obtained for all datasets (See additional file 1, Section 1: The full results of the robustness comparison of the Mutual Information measure to the Euclidean distance and to the Pearson correlation coefficient). Possible explanations for the domination of the MI-based scores on these datasets are given in the Discussion section. From the results it was impossible to distinguish between the Pearson correlation and the Euclidean distance, as expected from functionally-related scores. It is to be noted that the MI-based scores depicted in the above figures were generated by using histograms with 20 bins to estimate the pairwise MI between expression patterns. Nonetheless, the same conclusions regarding the superiority of the MI-based measures were obtained for a large range of different numbers of bins (between 10 to 30 bins) that comply with the heuristic rules mentioned in the Methods section.

### Experiment 2: Comparison of known clustering algorithms by the MI measure

In the above experiment we compared the robustness of the MI measure to that of the Pearson correlation and the Euclidean distance. Within the examined datasets, it was found that the MI measure is statistically superior to the conventional measures in the detection of clustering errors. Note that the comparison has been performed with respect to known final clustering solution *independently *of the compared clustering algorithms. In this experiment, we take one step ahead and compare the effectiveness of several known clustering algorithms. In particular, we grade the best solutions of the clustering algorithms by the MI-based homogeneity and separation scores and then compare it to the Pearson-based scores.

Evidently, the use of different clustering algorithms often results in different solutions. There is a large body of research that compares the performance of different clustering algorithms with respect to gene-expression levels (e.g., [11,26-28]). Nevertheless, these papers do not analyze the solutions by the MI-based measures, as we do here.

This experiment compares four clustering algorithms that have been widely applied to gene-expression patterns (referred to as the *elements *to be clustered). For the purpose of comparison, we selected several algorithms that are based on distant methodological concepts that are, except for one (the *sIB*), unrelated directly to information theory. Potentially, the MI measure could be integrated into each of the algorithms by some procedural modifications, leading to clustering solutions that better conform with the MI metric. Nevertheless, we prefer to use and analyze the conventional algorithmic forms as being used universally. To this end, our comparison of these algorithms does not indicate which one is better, but rather which one of them produces clustering solutions that are more confirmative with the MI-based measures. This purpose is particularly important in view of previous publications that concluded that there is a high degree of conformity between the three distance measures [16,17]. Apparently, despite this conformity, the implementation of these distance measures in different algorithms lead to diverse clustering solutions.

The four compared algorithms are the *K-means *[29], the *SOM *[30], the *Click *[31] and the *sIB *[32,33]. A comparison of part of these algorithms can be found in Gat-Viks et al. (2003) [21] and Shamir and Sharan (2002) [5].

#### The Yeast cell-cycle dataset

The study of the algorithms is based on their clustering solutions over the known dataset of yeast cell cycle [19], available at [34]. The dataset presents 72 experimental conditions of regulated yeast's genes whose transcript levels vary periodically within the cell cycle. The authors used yeast cultures that were synchronized by four independent methods: a factor arrest, elutriation, arrest of a cdc15 temperature-sensitive mutant, and arrest of a cdc28 temperature sensitive mutant (an additional 90 min data point in the cdc15 experiment was not used, as in Cho et al., 1998 [35]).

Using periodicity and correlation algorithms (e.g., Pearson correlation), the authors identified 800 genes as being periodically regulated. Note that although the yeast cell-cycle dataset has been extensively analyzed previously, its "correct" clustering is unknown. Spellman et al. (1998) [19] assumed that the expression patterns can be correlated to (somewhat arbitrary) five different profiles, representing genes known to be expressed in G1, S, G2, M, and the M/G1 stages, as indicated in the literature. Tamayo et al. (1999) [20], on the other hand, divided the genes into 30 clusters along with the identification of the correlations between genes that belong to different clusters. Shamir and Sharan (2002) [5] compared different clustering solutions with the same dataset based on 5, 6 and 7 clusters, as we do here.

The preparation of the dataset was performed in a similar manner to Gat-Viks et al. (2003) [21]. We used the expression profiles of genes which have up to three missing entries over the 72 conditions. The missing entries in each gene were completed with the average of its present entries. The use of row-average was implemented since it is the most commonly used method to treat missing data, despite the fact that other methods, such as *KNNimpute *and *SVDimpute*, were found to be more robust to missing value estimation [36].

#### Experimental results

The compared algorithms were tested with respect to the yeast cell-cycle dataset to obtain their best clustering solutions with 5, 6 and 7 clusters. The comparison between the algorithms was performed between solutions with the same number of clusters. Clustering solutions were graded by the MI-based homogeneity and separation scores (see Methods section). This grading method is conventionally used to determine the quality of a clustering solution when the true solution is unknown [5]. The MI measure was computed by using 6, 8 and 10 bins, in accordance with the proposed heuristics to define the number of bins for 72 conditions. It was found that the number of bins did not change the results of the comparison; hence, in the following we refer only to the results based on 8 bins.

**Figure 6:**
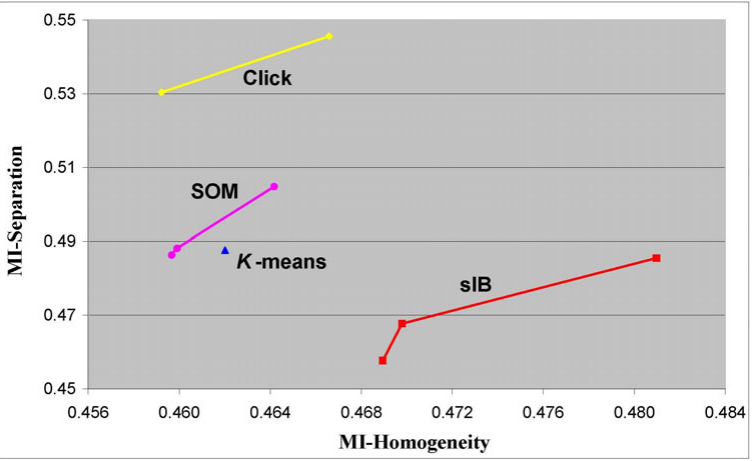
**MI-based scores of clustering solutions with 5 clusters**. Efficiency frontiers for solutions with 5 clusters, obtained by the *K-means*, the *SOM*, the *sIB *and the *Click *clustering algorithms. The clusters are obtained over the Yeast cell-cycle dataset with 800 genes and 72 experimental time-conditions. The scores are depicted on the MI-based Homogeneity-Separation plane.

**Figure 7:**
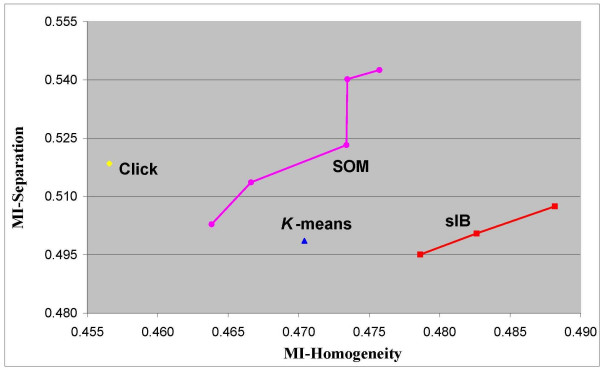
**MI-based scores of clustering solutions with 7 clusters**. Efficiency frontiers for solutions with 7 clusters, obtained by the *K-means*, the *SOM*, the *sIB *and the *Click *clustering algorithms. The clusters are obtained over the Yeast cell-cycle dataset with 800 genes and 72 experimental time-conditions. The scores are depicted on the MI-based Homogeneity-Separation plane.

The comparison results for 5 and 7 clusters are given in Figure 6 and Figure 7 respectively (similar results are obtained for 6 clusters – see section 2 in the additional appendix file). The axes indicate the MI-based homogeneity and separation scores. A clustering solution is considered better as the homogeneity score increases, while the separation score decreases. The figures present the best clustering. Thus, for each algorithm, the figures present only those solutions that are not dominated by any other solution of the same algorithm. In the case where there are several non-dominated clustering solutions per algorithm, these solutions form an *efficiency frontier *on the MI-based homogeneity-separation plane. This manner of evaluation of clustering solutions is accepted when the "true" clustering is unknown (e.g., [5]).

In general, we found that the *sIB *algorithm obtained the best solutions with respect to the MI-based homogeneity-separation scores for all the number of selected clusters: five clusters as shown in Figure 6; six clusters (presented in the additional appendix file); and 7 clusters as shown in Figure 7. All figures present at least one clustering solution of the *sIB *that dominates the best solutions that were obtained by the *K-means*, the *SOM *and the *Click *algorithms. The MI-based homogeneity scores of the *sIB *solutions in the efficiency frontier are significantly higher than those obtained by the other algorithms. In most cases, the same observation is also true with respect to the MI-based separation scores.

When considering *all *the clustering solutions (that are not necessarily part of the efficient frontier, and therefore are not necessarily presented in the figures) we found the following results (see Table 2). Eighty percent of the *sIB *solutions with 5 clusters have higher MI-based homogeneity scores with respect to the solutions obtained by the other algorithms. One hundred percent of the *sIB *solutions with 6 and 7 clusters have higher MI-based homogeneity scores with respect to the solutions obtained by the other algorithms. Thirty percent of the *sIB *solutions with 5, 6 clusters have lower MI-based separation scores with respect to the solutions obtained by the other algorithms. Forty eight percent of the *sIB *solutions with 7 clusters have lower MI-based separation scores with respect to the solutions obtained by the other algorithms. When analyzing all the obtained solutions with respect to both scores simultaneously, we found that 30% of the *sIB *solutions with 5 and 6 clusters have higher MI-based homogeneity and separation scores than those obtained by the other algorithms. Finally, for 5, 6 and 7 clusters' solutions there exists at least one *sIB *solution that has better MI-based homogeneity and separation (combined) scores with respect to all the solutions obtained by the other algorithms. Two of these solutions are the lower-left solutions in the *sIB *efficiency frontiers shown in Figure 6 and Figure 7.

**Table 2:** Comparative analysis of the *sIB *clustering solutions

No. of clusters	Better H. scores	Better S. scores	Better H/S Scores	Dominate solution
5	80%	30%	30%	Yes
6	100%	30%	30%	Yes
7	100%	48%	N/A	Yes

Comparative analysis of obtained clustering solutions in experiment 2 (not limited to the solutions in the efficiency frontier). The first column lists the number of clusters in the solutions. The second and third column give, respectively, the percentages of the sIB solutions that obtain a better MI-based Homogeneity and MI-based Separation scores with respect to the solutions of the other clustering algorithms (Click, K-means and SOM). The fourth column lists the percentages of the sIB solutions that obtain a better MI-based Homogeneity and Separation (combined) scores with respect to the solutions of the other clustering algorithms. The last column lists the existence of a sIB dominate solution over all the other solutions.

**Figure 8:**
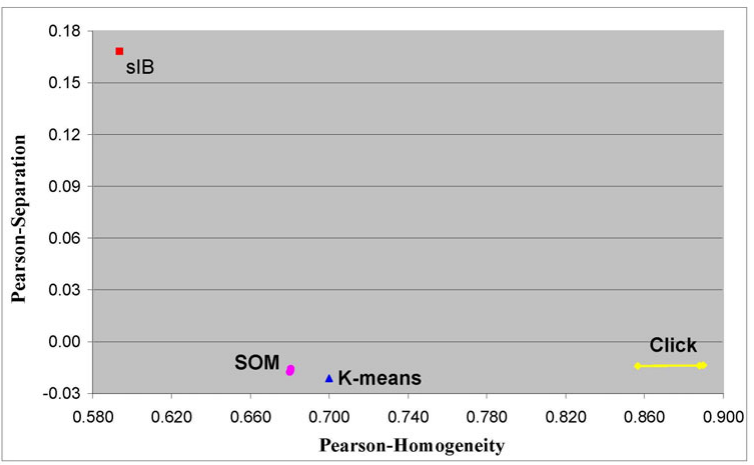
**Pearson correlation based scores of solutions with 5 clusters**. Efficiency frontiers for solutions with 5 clusters, obtained by the *K-means*, the *SOM*, the *sIB *and the *Click *clustering algorithms. The clusters are obtained over the Yeast cell-cycle dataset with 800 genes and 72 experimental time-conditions. The scores are depicted on the Pearson correlation based Homogeneity-Separation plane.

**Figure 9:**
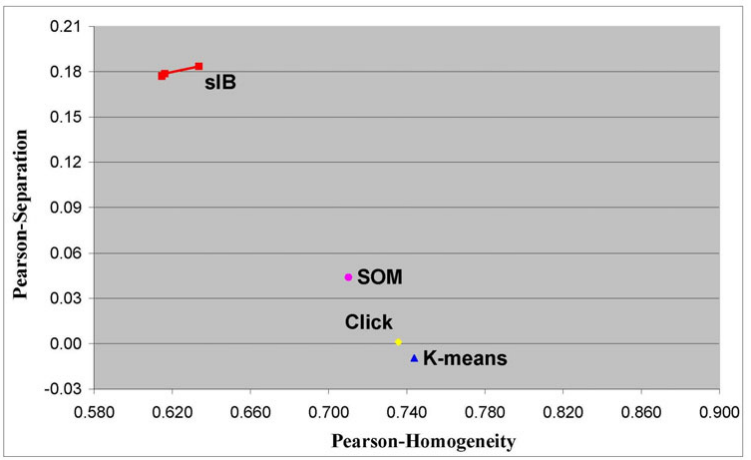
**Pearson correlation based scores of solutions with 7 clusters**. Efficiency frontiers for solutions with 7 clusters, obtained by the *K-means*, the *SOM*, the *sIB *and the *Click *clustering algorithms. The clusters are obtained over the Yeast cell-cycle dataset with 800 genes and 72 experimental time-conditions. The scores are depicted on the Pearson correlation based Homogeneity-Separation plane.

Note that when scoring the different algorithms with respect to the Euclidean or the Pearson measures, the (relative) ranking can be totally different. For example, Figure 8 and Figure 9 present respectively the efficiency frontier on the Pearson-based homogeneity-separation plane for solutions with 5 and 7 clusters that are obtained by the same four compared algorithms. Note that once the solutions are evaluated by a different distance measure, the ranking obtained is almost the opposite of the MI-based ranking. In this case, the *sIB *algorithm obtains the worst results relative to all the other algorithms. For 7-clusters solutions the *K*-*means *algorithm obtains the best solution. For 5-clusters solutions there is no clear dominate solution. However the *Click *solutions, which have the highest Homogeneity scores, obtain Separation scores that are very competitive to the solutions of the *SOM *and the *K-means*. These results emphasize once again that despite the conformity between the compared distance measures, as informed in the literature [16,17], further attention should be paid to the selection of a distance measure for analyzing the clustering of gene expression data.

## Discussion

This paper presents two related experiments. In the first experiment, which is based on known clustering solutions, we show the statistical superiority of the average MI-based measure independently of the selected clustering algorithm. In the second experiment, we show that the use of different distance measures can yield very different results when evaluating the solutions of known clustering algorithms. This is particularly true when looking at the score of the "best clustering solution" rather than the averaged score of a group of solutions, as we did in Experiment 1. This important fact is often overlooked in the literature. The essential question "which distance measure to use?" remains without a definitive answer, yet, in view of the first experiment, we propose to further investigate it by integrating the MI-based distance measure into known clustering algorithms.

The statistical superiority of the MI-based score in the first experiment can be attributed to several appealing properties of this measure, as elaborated in the Methods section. The first property is the use of MI as a generalized measure of correlation between variables, a property which seems valuable for gene expression data [15-17]. The second property is the robustness of the MI measure with respect to missing expression values [46]. Finally, our use of equal-probability bins to estimate the MI score provides considerable protection against outlier, since the contributions of all expression values within a bin to this estimation are identical, regardless of their actual values.

We can indicate two possible reasons why the *sIB *algorithm outperforms the other clustering algorithms with respect to the MI-based homogeneity and separation scores. The first reason is the nature of the stochastic transition matrix (STM) which is used by the *sIB *as an input. Although the STM is generated from a conventional pairwise similarity matrix, once it is formed it represents the (dis)similarity relations between *all *the patterns in the dataset. Based on the STM, the *sIB *associates an expression profile to a cluster by using the joint probability distribution of *all *the expression patterns in the data. This is a possible explanation as to why the *sIB *generates solutions with higher MI-based homogeneity score than those obtained by the other algorithms that rely only on the pairwise distances, or only on the patterns in the particular associated cluster. Moreover, the construction of a joint probability distribution that reflects the associations between *all *the patterns in the dataset provides an additional normalization procedure that naturalizes external effects, such as measuring and experimental errors.

The second possible reason for the superiority of the *sIB *is evidently the use of MI in its objective function. The *sIB *aims at minimizing the loss of information on the gene-expression profiles during the clustering process. The algorithm objective is to find a clustering solution that (locally) maximizes the mutual information between clusters and a relevance variable which is associated with the gene patterns [32]. Note, however, that the use of MI in the *sIB *objective function is considerably different than the use of MI when computing the homogeneity and separation scores. The former is based on the joint distribution of all gene patterns, while the latter is based on the joint distribution between each gene and its associated cluster. Moreover, our way to derive the joint distribution of patterns and clusters (by a two-dimensional histogram of empirically expression levels) is very different than the exponential transformation used by the *sIB *to obtain the joint distribution of patterns. Nevertheless, it seems that although these definitions and calculations are different, they are sufficiently associated such that the *sIB *produces a clustering solution with high MI-based scores.

## Conclusion

In this work we analyze the performance of the Mutual Information (MI) distance measure for clustering of gene-expression data. In comparison to the Pearson correlation and the Euclidean distance, the MI measure is known to have some advantages: it is a generalized measure of statistical dependence in the data, and it is reasonably immune against missing data and outliers. In this work we show that the average MI measure also yields a higher power of test among different clustering solutions, thus, this measure is potentially more robust for differentiating erroneous clustering solutions. A comparative study of known clustering algorithms reveals that their best solutions are ranked almost oppositely when using different distance measures. In view of these results, further attention should be paid to the selection of a proper distance measure for the evaluation of clustering of gene expression data. One future direction is the integration of the MI-measure in known clustering algorithms. Another potential research is to implement Daub et al. (2004) [18] methods in the robustness analysis.

## Methods

### Similarity measures

This section discusses three similarity measures and their properties: the *Euclidean distance *and the *Pearson correlation *that are commonly used measures in gene expression clustering [6,37-39] and the proposed *mutual information *(MI) measure. These measures quantify a pairwise distance between expression profiles over *n *conditions that are represented by the two vectors **x **= (*x*_1_,..., *x*_*n*_) and **y **= (*y*_1_,..., *y*_*n*_).

#### Euclidean Distance and Pearson Correlation

The Euclidean distance between two expression profiles is given by

E(x,y)=∑i=1n(xi−yi)2.     (1)
 MathType@MTEF@5@5@+=feaafiart1ev1aaatCvAUfKttLearuWrP9MDH5MBPbIqV92AaeXatLxBI9gBaebbnrfifHhDYfgasaacH8akY=wiFfYdH8Gipec8Eeeu0xXdbba9frFj0=OqFfea0dXdd9vqai=hGuQ8kuc9pgc9s8qqaq=dirpe0xb9q8qiLsFr0=vr0=vr0dc8meaabaqaciaacaGaaeqabaqabeGadaaakeaacqWGfbqrcqGGOaakieqacqWF4baEcqGGSaalcqWF5bqEcqGGPaqkcqGH9aqpdaGcaaqaamaaqadabaGaeiikaGIaemiEaG3aaSbaaSqaaiabdMgaPbqabaGccqGHsislcqWG5bqEdaWgaaWcbaGaemyAaKgabeaakiabcMcaPmaaCaaaleqabaGaeGOmaidaaaqaaiabdMgaPjabg2da9iabigdaXaqaaiabd6gaUbqdcqGHris5aaWcbeaakiabc6caUiaaxMaacaWLjaWaaeWaaeaacqaIXaqmaiaawIcacaGLPaaaaaa@4994@

It measures similarity according to positive linear correlation between expression profiles, which may identify similar or identical regulation [6]. The measure is highly influenced by the magnitude of changes in the measured expression profiles. Therefore, it should be used mainly for expression data that are suitably normalized. When such normalization is used, the Euclidean distance and the Pearson correlation are monotonically related, as indicated below.

Numerous biological researches (e.g., [17,28,40,41]) implemented the Euclidean distance as a similarity measure for gene expression analysis. Most of these publications analyzed similar expression trends, i.e., simultaneous up-regulated or down-regulated expression levels. From a biological viewpoint, a relative up/down-regulation of gene expressions is often considered more important than the amplitude absolute changes [28].

The Pearson correlation coefficient between two expression patterns (e.g., [1,10-14]) is defined as

R(x,y)=∑i=1n(xi−x¯)⋅(yi−y¯)∑i=1n(xi−x¯)2∑i=1n(yi−y¯)2,     (2)
 MathType@MTEF@5@5@+=feaafiart1ev1aaatCvAUfKttLearuWrP9MDH5MBPbIqV92AaeXatLxBI9gBaebbnrfifHhDYfgasaacH8akY=wiFfYdH8Gipec8Eeeu0xXdbba9frFj0=OqFfea0dXdd9vqai=hGuQ8kuc9pgc9s8qqaq=dirpe0xb9q8qiLsFr0=vr0=vr0dc8meaabaqaciaacaGaaeqabaqabeGadaaakeaacqWGsbGucqGGOaakieqacqWF4baEcqGGSaalcqWF5bqEcqGGPaqkcqGH9aqpdaWcaaqaamaaqadabaGaeiikaGIaemiEaG3aaSbaaSqaaiabdMgaPbqabaGccqGHsislcuWG4baEgaqeaiabcMcaPaWcbaGaemyAaKMaeyypa0JaeGymaedabaGaemOBa4ganiabggHiLdGccqGHflY1cqGGOaakcqWG5bqEdaWgaaWcbaGaemyAaKgabeaakiabgkHiTiqbdMha5zaaraGaeiykaKcabaWaaOaaaeaadaaeWaqaaiabcIcaOiabdIha4naaBaaaleaacqWGPbqAaeqaaOGaeyOeI0IafmiEaGNbaebacqGGPaqkdaahaaWcbeqaaiabikdaYaaaaeaacqWGPbqAcqGH9aqpcqaIXaqmaeaacqWGUbGBa0GaeyyeIuoakmaaqadabaGaeiikaGIaemyEaK3aaSbaaSqaaiabdMgaPbqabaGccqGHsislcuWG5bqEgaqeaiabcMcaPmaaCaaaleqabaGaeGOmaidaaaqaaiabdMgaPjabg2da9iabigdaXaqaaiabd6gaUbqdcqGHris5aaWcbeaaaaGccqGGSaalcaWLjaGaaCzcamaabmaabaGaeGOmaidacaGLOaGaayzkaaaaaa@6ED9@

where x¯
 MathType@MTEF@5@5@+=feaafiart1ev1aaatCvAUfKttLearuWrP9MDH5MBPbIqV92AaeXatLxBI9gBaebbnrfifHhDYfgasaacH8akY=wiFfYdH8Gipec8Eeeu0xXdbba9frFj0=OqFfea0dXdd9vqai=hGuQ8kuc9pgc9s8qqaq=dirpe0xb9q8qiLsFr0=vr0=vr0dc8meaabaqaciaacaGaaeqabaqabeGadaaakeaacuWG4baEgaqeaaaa@2E3D@, y¯
 MathType@MTEF@5@5@+=feaafiart1ev1aaatCvAUfKttLearuWrP9MDH5MBPbIqV92AaeXatLxBI9gBaebbnrfifHhDYfgasaacH8akY=wiFfYdH8Gipec8Eeeu0xXdbba9frFj0=OqFfea0dXdd9vqai=hGuQ8kuc9pgc9s8qqaq=dirpe0xb9q8qiLsFr0=vr0=vr0dc8meaabaqaciaacaGaaeqabaqabeGadaaakeaacuWG5bqEgaqeaaaa@2E3F@ denote the average patterns level.

The Pearson correlation reflects the degree of linear relationship between two patterns. It ranges between -1 to +1, reflecting respectively a perfect negative (positive) linear relationship between the patterns. A zero correlation value implies that there is no linear relationship between the two patterns, yet it gives no indication regarding nonlinear relationships that might exist between the patterns.

The correlation coefficient is invariant under any scalar transformation of the data. Accordingly, two expression profiles that have "identical" shapes with different magnitudes will obtain a correlation value of 1. The ability to measure (dis)similarities according to positive and negative correlations can help to identify control processes that antagonistically regulate downstream pathways [6]. Nonetheless, the majority of the publications utilize only the positive correlation range, while others map the entire range of the correlation coefficient to obtain values between 0 and 1.

Gene expression measurements, like other empirical measurements, suffer from noise effects. Variations in the measurements might come from many sources: intrachip defects, variation within a single lot of chips, variation within an experiment, and biological variation for a particular gene [42]. Both the Pearson correlation and the Euclidean distance are sensitive to noise effects and outliers. A single outlier could transform the Euclidean distance to an unbounded value, while transforming the Pearson correlation to any value between -1 and 1 [43]. Both measures are easily distorted when the expression levels are not uniformly distributed across the expression pattern. For example, two expression patterns with one high measured value at the same cellular condition will obtain a high correlation coefficient score, regardless of the expression values of the other cellular conditions [44]. Similarly, a large difference in a single expression level at the same cellular condition will lead to a high Euclidean distance, regardless of the other expression levels. In this way, outlying points can bias the Correlation coefficient and the Euclidean distance.

Both the Pearson correlation and the Euclidean distance require complete gene expression profiles as input. However, gene-expression microarray experiments often generate datasets with missing expression values. Therefore, another source of uncertainty when implementing these measures is the need to use methods for estimating missing data, such as *row average *or *singular value decomposition *[26,36].

#### Mutual Information

The Mutual *information *(MI) provides a general measure for dependencies in the data, in particular, positive, negative and nonlinear correlations. It is a well known measure in *information theory *[45] that has been used to analyze gene-expression data [6,16,17,44,46]. The used MI measure requires the expression patterns to be represented by discrete random variables. Given two random variables *X*, *Y *with respective ranges *x*_*i *_∈ *A*_*x*_, *y*_*j *_∈ *A*_*j *_and probability distributions functions *P*(*X *= *x*_*i*_) ≡ *p*_*i*_, *P*(*Y *= *y*_*j*_) ≡ *p*_*j*_, the Mutual information between two expression patterns, represented by random variables *X *and *Y*, is given by

I(X;Y)=∑i∑jpijlog⁡pijpipj.     (3)
 MathType@MTEF@5@5@+=feaafiart1ev1aaatCvAUfKttLearuWrP9MDH5MBPbIqV92AaeXatLxBI9gBaebbnrfifHhDYfgasaacH8akY=wiFfYdH8Gipec8Eeeu0xXdbba9frFj0=OqFfea0dXdd9vqai=hGuQ8kuc9pgc9s8qqaq=dirpe0xb9q8qiLsFr0=vr0=vr0dc8meaabaqaciaacaGaaeqabaqabeGadaaakeaacqWGjbqscqGGOaakcqWGybawcqGG7aWocqWGzbqwcqGGPaqkcqGH9aqpdaaeqaqaamaaqababaGaemiCaa3aaSbaaSqaaiabdMgaPjabdQgaQbqabaaabaGaemOAaOgabeqdcqGHris5aaWcbaGaemyAaKgabeqdcqGHris5aOGagiiBaWMaei4Ba8Maei4zaC2aaSaaaeaacqWGWbaCdaWgaaWcbaGaemyAaKMaemOAaOgabeaaaOqaaiabdchaWnaaBaaaleaacqWGPbqAaeqaaOGaemiCaa3aaSbaaSqaaiabdQgaQbqabaaaaOGaeiOla4IaaCzcaiaaxMaadaqadaqaaiabiodaZaGaayjkaiaawMcaaaaa@51DB@

The MI is always non-negative. It equals zero if and only if *X *and *Y *are statistically independent, meaning that *X *contains no information about *Y *and vice versa. A zero MI indicates that the patterns do not follow *any kind *of dependence, an indication which is impossible to obtain from the Pearson correlation or the Euclidean distance [15]. This property makes the MI a generalized measure of correlation, which is advantageous in gene expression analysis. For example, if one gene acts as a transcription factor only when it is expressed at a midrange level, then the scatter plot between this transcription factor and the other genes might closely resemble a normal distribution rather than a linear model. The Pearson correlation coefficient under this scenario will obtain a low score, while the MI measure can obtain a high score [44].

Another important feature of the MI is its robustness with respect to missing expression values. In fact the MI can be estimated from datasets of different sizes. This is advantageous in analyzing expression datasets that contain a certain amount (up to 25%) of missing values [46].

The MI between a pair of expression patterns is upper bounded by their marginal entropies. Accordingly, the MI measure exhibits a low value if the marginal entropies are low, even if the patterns are completely correlated. Therefore, there is a need to normalize the MI measure, giving a high score for highly correlated sequences, independent of their marginal entropies. There are several ways to carry out such normalization. Michaels et al. (1998) [17] normalize the MI measure by dividing it by the maximal marginal entropy of the considered sequence. Steuer et al. (2002) [16] suggest a rank-ordering procedure. We use a partitioning method for equal-probability bins, where each bin contains approximately the same number of data points. The width of each bin is determined by the local density of the measured expression levels. Besides the obtained normalization, the proposed method is advantageous also in terms of outlier protection. The MI treats each expression level equally, regardless of the actual value, and thus is less biased by outliers.

As noted above, the use of the discrete form of the MI measure requires the discretization of the continuous expression values. The most straightforward and commonly used discretization technique is to use a histogram-based procedure [16,18,44]. We use a two-dimensional histogram to approximate the joint probability density function of two expression patterns. We use the same number of bins for all expression patterns. However, the bins in each expression pattern are determined independently according to the density of the expression values. The joint probabilities are then estimated by the corresponding relative frequencies of expression values in each bin in the two-dimensional histogram. This estimation requires the sorting of expression values with a computational complexity of *O*(*n*log*n*), where *n *is the number of expression values. Such sorting is not required when calculating the Pearson coefficient or the Euclidean distance measure. The number of bins should be moderate enough to allow good estimates of the probability function. If this number is too small or too large, then all bins will contain approximately the same number of expression values. In such a case the joint distributions of all pairs of expression patterns will be similar and will lead to the same MI value. There is no optimal solution to choose the number of bins, since it depends on data normalization and on the particular biological application [18]. Consequently, the number of bins is often obtained heuristically. We follow Sturges (1926) [47] and Law and Kelton (1991) [48] and use the following simple lower/upper bounds on the number of bins:

*M*_*l *_= ⌊1 + log_2_*n*⌋ and *M*_*u *_= n
 MathType@MTEF@5@5@+=feaafiart1ev1aaatCvAUfKttLearuWrP9MDH5MBPbIqV92AaeXatLxBI9gBaebbnrfifHhDYfgasaacH8akY=wiFfYdH8Gipec8Eeeu0xXdbba9frFj0=OqFfea0dXdd9vqai=hGuQ8kuc9pgc9s8qqaq=dirpe0xb9q8qiLsFr0=vr0=vr0dc8meaabaqaciaacaGaaeqabaqabeGadaaakeaadaGcaaqaaiabd6gaUbWcbeaaaaa@2E2C@. In the Results section we show that within this range for the number of bins, the MI measure outperforms the other distance measures.

### Assessment of clustering quality

The homogeneity and the separation functions are often used to determine the quality of a clustering solution when the true solution is unknown [5]. When considering similarity measures like the MI and the Pearson correlation, high homogeneity implies that elements in the same cluster are very similar to each other; while low separation implies that elements from different clusters are very dissimilar to each other. The two criteria are widely used in gene expression analysis, as well as in other fields [12,21,28,31,39,49].

Consider a set of *N *elements (genes or samples represented by expression patterns or profiles) X ≡ {*X*_1_, *X*_2_, ..., *X*_*N*_} divided into *k *clusters. Denote by *X*_*i *_and *C*(*X*_* i*_) the expression pattern of element *i *and the expression pattern of its cluster respectively, then the homogeneity is given by

Hm=1N∑Xi∈XD(Xi,C(Xi))     (4)
 MathType@MTEF@5@5@+=feaafiart1ev1aaatCvAUfKttLearuWrP9MDH5MBPbIqV92AaeXatLxBI9gBaebbnrfifHhDYfgasaacH8akY=wiFfYdH8Gipec8Eeeu0xXdbba9frFj0=OqFfea0dXdd9vqai=hGuQ8kuc9pgc9s8qqaq=dirpe0xb9q8qiLsFr0=vr0=vr0dc8meaabaqaciaacaGaaeqabaqabeGadaaakeaacqWGibascqWGTbqBcqGH9aqpdaWcaaqaaiabigdaXaqaaiabd6eaobaadaaeqbqaaiabdseaejabcIcaOiabdIfaynaaBaaaleaacqWGPbqAaeqaaOGaeiilaWIaem4qamKaeiikaGIaemiwaG1aaSbaaSqaaiabdMgaPbqabaGccqGGPaqkcqGGPaqkaSqaaiabdIfaynaaBaaameaacqWGPbqAaeqaaSGaeyicI4mcbeGae8hwaGfabeqdcqGHris5aOGaaCzcaiaaxMaadaqadaqaaiabisda0aGaayjkaiaawMcaaaaa@49CA@

where *D*(·) represents a given similarity measure, i.e., the Euclidean distance, the Pearson correlation or the Mutual Information. The solution separation score is evaluated by the weighted average similarity between cluster expression patterns: denote the expression patterns of clusters *t*_1_,...,*t*_*k*_, by *C*_*t1*_,...,*C_tk_*,  then, the average separation is given by

Sp=∑i≠jNiNjD(Cti,Ctj)/∑i≠jNiNj,     (5)
 MathType@MTEF@5@5@+=feaafiart1ev1aaatCvAUfKttLearuWrP9MDH5MBPbIqV92AaeXatLxBI9gBaebbnrfifHhDYfgasaacH8akY=wiFfYdH8Gipec8Eeeu0xXdbba9frFj0=OqFfea0dXdd9vqai=hGuQ8kuc9pgc9s8qqaq=dirpe0xb9q8qiLsFr0=vr0=vr0dc8meaabaqaciaacaGaaeqabaqabeGadaaakeaacqWGtbWucqWGWbaCcqGH9aqpdaaeqbqaamaalyaabaGaemOta40aaSbaaSqaaiabdMgaPbqabaGccqWGobGtdaWgaaWcbaGaemOAaOgabeaakiabdseaejabcIcaOiabdoeadnaaBaaaleaacqWG0baDdaWgaaadbaGaemyAaKgabeaaaSqabaGccqGGSaalcqWGdbWqdaWgaaWcbaGaemiDaq3aaSbaaWqaaiabdQgaQbqabaaaleqaaOGaeiykaKcabaWaaabuaeaacqWGobGtdaWgaaWcbaGaemyAaKgabeaakiabd6eaonaaBaaaleaacqWGQbGAaeqaaaqaaiabdMgaPjabgcMi5kabdQgaQbqab0GaeyyeIuoaaaaaleaacqWGPbqAcqGHGjsUcqWGQbGAaeqaniabggHiLdGccqGGSaalcaWLjaGaaCzcamaabmaabaGaeGynaudacaGLOaGaayzkaaaaaa@5951@

where *N*_*i*_, *N*_*j *_are the number of elements in cluster *t*_*i*_, *t*_*j *_respectively. The homogeneity and the separation are typically conflicting functions – usually the better is the homogeneity of a solution, the worse is its separation, and vice versa.

### Known Clustering Algorithms

The underlying concepts and the parameters of each of the clustering algorithms are given in an additional file 1, section 2. The *K-means *was implemented by Matlab procedures. The SOM algorithm was implemented by *GeneCluster 2.0 *[20,49]. The *Click *was implemented by *Expander *[50,51]. The *sIB *was implemented by *IBA_1.0 *[52]. Further information regarding common unsupervised clustering and learning methods can also be found in [53-55].

## Authors' contributions

IP participated in the design of the study, produced the necessary data and software, carried out the analyses and drafted the manuscript. IBG conceived the study, designed and supervised the work, helped to analyze the results and drafted the manuscript. OM helped in the research coordination and review. The authors read and approved the final manuscript.

## Supplementary Material

Additional file 1 Used software and parameters for comparison of clustering algorithms. The file contains the following 2 sections. Section 1: A comparison study of the *Mutual Information *(MI) measure, the Euclidean distance and the Pearson correlation coefficient. The robustness comparison is performed by using four public gene expression datasets. Each dataset contains two types of samples with a clear biological distinction, leading to a 'true' bi-clustering solution. Section 2: Details of the underlying concepts and parameters of the four clustering algorithms that were used in experiment 2. Additionally, the full results of the algorithms comparison are presented. Click here for file
