# Supplementary material for: Evaluation of gene-expression clustering via mutual information distance measure
Source: BMC Bioinformatics. 2007 Mar 30;8:111. doi: 10.1186/1471-2105-8-111 (PMC1858704; doi:10.1186/1471-2105-8-111)
Supplement: Additional file 1 — Used software and parameters for comparison of clustering algorithms. The file contains the following 2 sections. Section 1: A comparison study of the Mutual Information (MI) measure, the Euclidean distance and the Pearson correlation coefficient. The robustness comparison is performed by using four public gene expression datasets. Each dataset contains two types of samples with a clear biological distinction, leading to a 'true' bi-clustering solution. Section 2: Details of the underlying concepts and parameters of the four clustering algorithms that were used in experiment 2. Additionally, the full results of the algorithms comparison are presented. [file 1471-2105-8-111-S1.pdf]

USED SOFTWARE AND PARAMETERS FOR COMPARISON OF CLUSTERING ALGORITHMS

(Technical appendix to Priness Maimon and Ben-Gal 2006)[1]

By
Ido Priness and Irad Ben-Gal
Department of Industrial Engineering
Tel-Aviv University
Israel, 69778

Abstract

This paper provides details on the results described in Priness and Ben-Gal (2006). The paper presents the results of the robustness comparison of the *Mutual Information* (MI) measure to the Euclidean distance and to the Pearson correlation coefficient. In addition, it shortly outlines the underlying concepts and parameters of four clustering algorithms that were evaluated in Section 4 in Priness and Ben-Gal (2006). The four compared algorithms are the K-means (Mac-Queen, 1965), the SOM (Kohonen, 1997), the Click (Sharan and Shamir, 2000) and the sIB (Tishby and Slonim, 2000, Slonim, 2002). The description of the algorithms is based on Shamir and Sharan (2002) and Slonim (2002). The parameters that were used in each of the algorithms are given in the tables at the end of the paper.

1. Robustness Comparison

The robustness comparison was performed using four public gene expression datasets. Each dataset contains two types of samples with a clear biological distinction, leading to a 'true' bi-clustering solution. The comparison results are presented henceforth by 4 couple of figures. The details on the computation of the MI measure are given next.

[1] Priness, Maimon and Ben-Gal (2006), Robust Gene-Expression Clustering via Mutual Information Distance Measure, accepted to *BMC Bioinformatics*.

Data set 1: 28 lung cancer samples Vs. 23 colon cancer samples

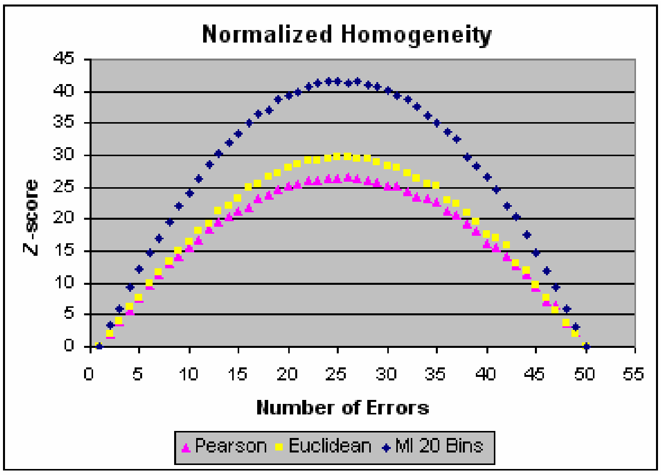

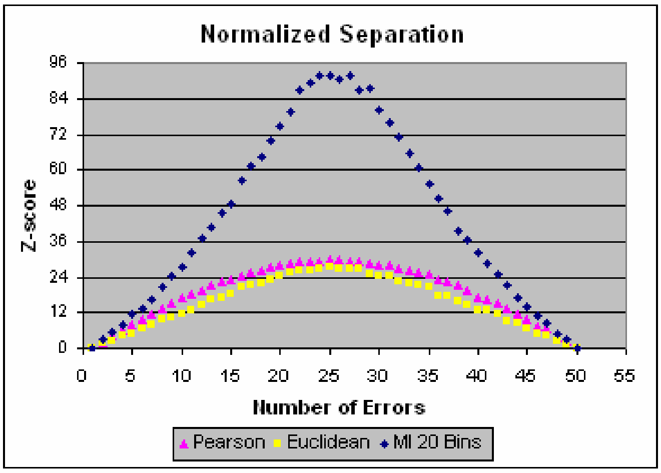

Data set 2: 26 breast cancer samples Vs. 28 lung cancer samples

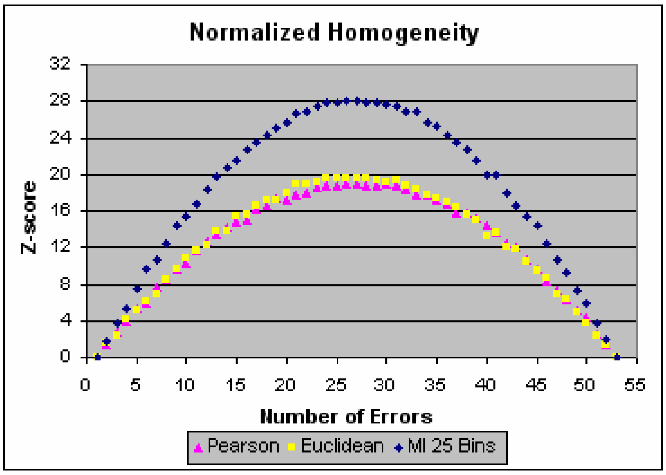

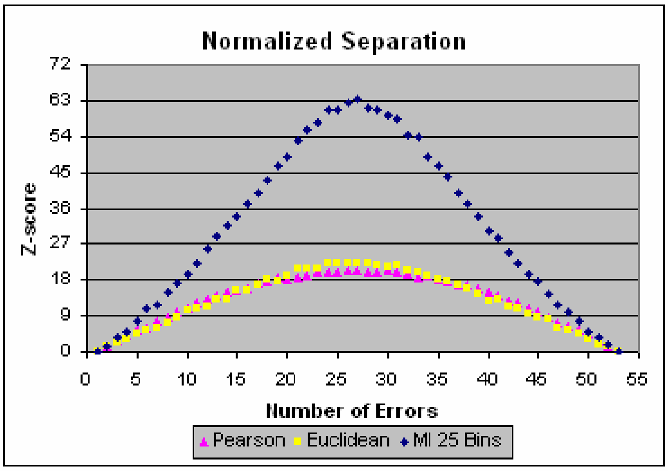

Data set 3: 26 breast cancer samples vs. 23 colon cancer samples

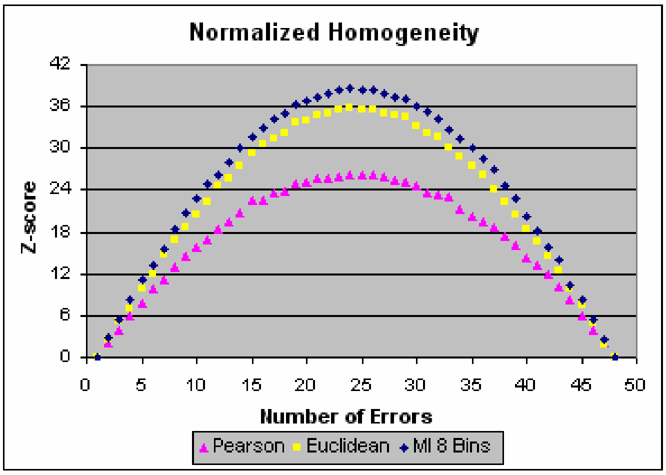

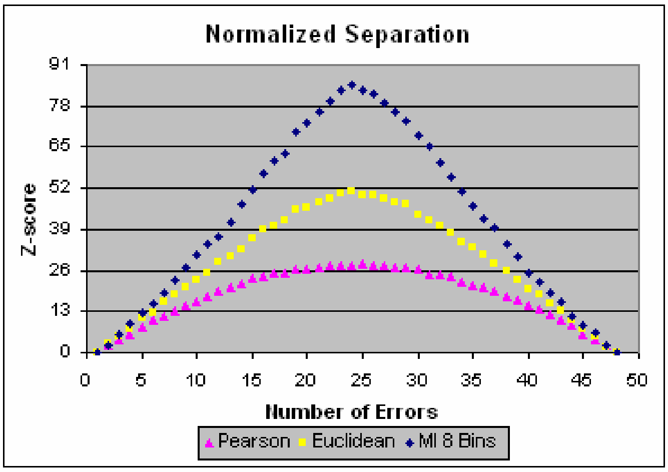

Data set 4: 40 colon cancer samples vs. 22 normal colon samples

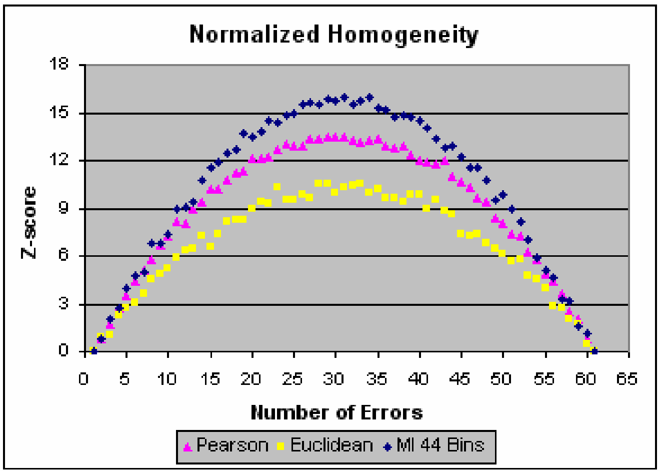

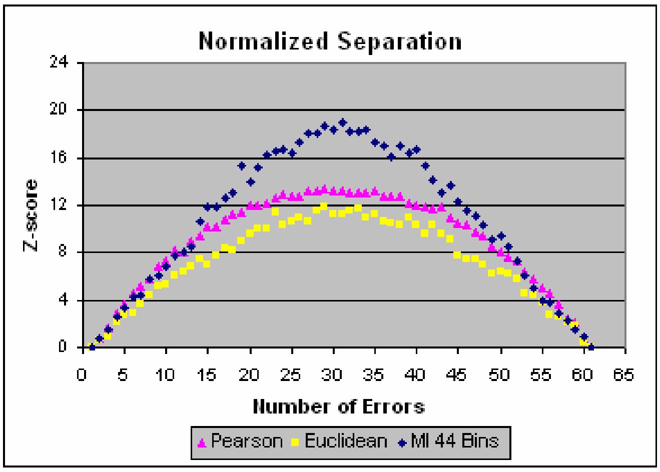

As noted in the paper, the use of the discrete form of the MI measure requires the discretization of the continuous expression values. A straightforward discretization technique is to use a histogram-based procedure (Steuer et al., 2002, Daub et al, 2004). We use a two-dimensional histogram to approximate the joint probability density function of two expression patterns. We use the same number of bins for all expression patterns. However, the bins in each expression pattern are determined independently according to the density of the expression values. The joint probabilities are then estimated by the corresponding relative frequencies of expression values in each bin in the two-dimensional histogram. The number of bins should be moderate enough to allow good estimates of the probability function. If this number is too small or too large, then all bins will contain approximately the same number of expression values. In such a case, the joint distributions of all pairs of expression patterns will be similar and will lead to the same MI value. There is no optimal solution to choose the number of bins, since it depends on data normalization and on the particular biological application (Daub et al., 2004). Consequently, the number of bins is often obtained heuristically. We follow Sturges (1926) and Law and Kelton (1991) and use the following simple lower / upper bounds on the number of bins:
$M_l = \lfloor 1 + \log_2 n \rfloor$ and $M_u = \sqrt{n}$. In Section 3 we show that within this range for the number of bins, the MI measure outperforms the other distance measures.

2. Algorithms Evaluation

This section compares four clustering algorithms that have been widely applied to gene-expression patterns (referred to as the *elements* to be clustered).
The four compared algorithms are: i) the *K-means* (MacQueen, 1965); ii) the *SOM* (Kohonen, 1997); iii) the *Click* (Sharan and Shamir, 2000); and iv) the *sIB* (Tishby and Slonim, 2000).
The *K-means* was implemented by Matlab procedures. The SOM algorithm was implemented by *GeneCluster 2.0* (Tamayo et al., 1997) available at http:// www.broad.mit.edu/cancer/software/genecluster2/gc2.html. The *Click* was implemented by *Expander* (Sharan and Shamir, 2000), available at

http://www.cs.tau.ac.il/~rshamir/ expander/expander.html. The *sIB* was implemented by *IBA_1.0* (Slonim, 2005), available at http://www.princeton.edu/~nslonim/

Further information regarding common unsupervised clustering and learning methods can also be found in Everitt (1993), Mirkin (1996) and Hansen and Jaumard (1997)

In the following we give details on the computation of each method.

2.1. *K-means*

K-means (MacQueen, 1967) is one of the simplest unsupervised learning algorithms. The goal is to divide the objects into K clusters such that the homogeneity score, which is calculated relatively to the centroids of the clusters, is minimized. The algorithm is composed of the following steps (based on Shamir and Sharan , 2002):

Phase 1:

1. Start with arbitrary *k* elements *(gene expression patterns*) as the clusters *centroid*s.
2. Assign each remaining element *i* to the cluster which *has the closest centroid*.
3. Compute modified cluster centers and the cumulative distance measure between each centroid and its assigned elements.
4. **If** the cumulative distance measure does not grow and at least one element moved **then** go to step 2. Otherwise start phase 2.

Phase 2:

1. For each element *i* do: Assign the element to the cluster whose centroid is the closest and compute modified cluster centroids.
2. **If** at least 1 element moved during step 1 **then** repeat step 1. Otherwise **stop**.

In the *K-means* algorithm we performed 500 random initiations of cluster centroids for each number of required clusters (5, 6 and 7). Then, we selected the best solution among all clustering solutions with the same number of clusters. The K-means was implemented by *Matlab* procedures

2.2. Self Organizing Maps (SOM)

Self Organizing Maps were developed by Kohonen (1997) relying on the assumption that the number of clusters is known. Those clusters are organized as a set of nodes in a hypothetical "elastic" network, with a simple neighborhood structure such as a two-dimensional grid. Each of these nodes is associated with a reference vector in R^n and each element is associated with one of the reference vectors.

The initial positioning of nodes is selected at random. The algorithm iteratively picks a random element, identifies the nearest reference node, and updates the reference nodes according to a learning function $\tau(\cdot)$. The learning function affects more those nodes that are closer to the element, and decreases with the iteration number.

The algorithm is composed of the following steps (Shamir and Sharan, 2002):

Denote by $f_i(k)$ the position of node *k* at the *i*th iteration

Arbitrarily set the reference vectors $f_1(k) \in R^A$ for each node *k*.

For $i = 1$ until no node location is changed by more than ε **do**:

- Randomly pick element *x*.
- Find the node k_x with reference vector $f_i(k)$ closest to *x*.
- Update all reference vectors: $f_{i+1}(k) = f_i(k) + \tau(D(k, k_x), i)[x - f_i(k)]$, where $D(k, k_x)$ denotes the Euclidean distance between a node and the reference vector of the element.

Assign each element to the cluster with the closest reference vector.

We denote by $N_i(k_x)$ the set of nodes within a radius distance σ_i from node k_x. Consequently, the used "Neighborhood" learning function $\tau(D(k, k_x), i) = \alpha_i$ iff $k \in N_i(k_X)$. Otherwise we used $\tau(D(k, k_x), i) = 0$. Thus, α_i determines the learning "rate", and σ_i determines which of the nodes will be updated. Both parameters decrease in *i*.

The SOM algorithm was implemented by *GeneCluster 2.0* (see Tamayo et al., 1999). This is a clustering software that implements the SOM algorithm and available at http://waldo.wi.mit.edu/MPR. In the experiment we used $\alpha_i = \alpha_{init}\left(\alpha_{fin}/\alpha_{init}\right)^{i/F}$, where α_{init} and α_{fin} denote the learning rate in the first iteration and the last iteration respectively. F is the maximal number of allowed iterations and equals 50,000.

For solutions with 5 and 7 clusters the structure of the network was 1×5 and 1×7 respectively. For solutions with 6 clusters the analyzed network structures were 1×6 and 2×3. The set $N_i(k_x)$ included all the nodes within a radius distance $\sigma_i = \sigma_{init}\left(\sigma_{fin}/\sigma_{init}\right)^{i/F}$ from k_x, where σ_{init} and σ_{fin} denote the radius in the first iteration and the last iteration respectively. In addition to the default values of *GeneCluster2.0* the analyzed combinations of parameters included $\alpha_{init} \in$[0.05, 0.3] with steps of 0.05, α_{fin}=0.005, $\sigma_{init} \in$[5, 10] and σ_{fin}=0.5.

2.3. Click

The *Click* algorithm (Cluster Identification via Connectivity Kernels) was proposed by Sharan and Shamir (2000). It is based on a probabilistic graph theory model. The underlying assumption in the model is that pairwise similarity values between elements are normally distributed. Whereas similar values between mates (i.e., elements that belong to the same cluster) are normally distributed with mean μ_T and variance σ_T^2. Those values between non-mates follow the same distribution with mean μ_F, where $\mu_F > \mu_T$, and variance σ_F^2. The model is represented by a weighted similarity graph, where the elements are represented by the nodes. The probability that two elements, i and j, are mates is reflected by the weight of the connecting edge w_{ij}. This weight is computed by the following log-likelihood ratio which is based on the normality assumption:

$$w_{ij} = \ln \frac{\Pr(i, j \text{ are mates} \mid D(i,j))}{\Pr(i, j \text{ are non-mates} \mid D(i,j))} = \ln \frac{p\sigma_F}{(1-p)\sigma_T} + \frac{(D(i,j) - \mu_F)^2}{2\sigma_F^2} - \frac{(D(i,j) - \mu_T)^2}{2\sigma_T^2},$$

where $D(i,j)$ is the input distance measure between the two elements and *p* reflects the *a-priori* probability that two randomly chosen elements are mates. The parameters μ_T, μ_F, σ_T^2, σ_F^2, and *p* can be computed directly from a given clustering solution or estimated by using the EM algorithm (Shamir and Sharan, 2002). *Click* identifies highly connected subgraphs as clusters. It iteratively applies a minimum edge cut to the remaining subgraph. The subgraph is determined to be a cluster if the value of a minimum cut therein is positive. This condition is met if and only if for every cut in the subgraph the probability that it contains only edges between mates exceeds the probability that it contains only edges between non-mates.

In this work we used the software tool *Expander* which applies the *Click* algorithms. *Expander* is described in Sharan et al. (2003) and available at www.cs.tau.ac.il/~rshamir/expander/expander.html. In addition to gene expression data, the software gets as an input also a threshold value for the average required homogeneity. This threshold is used to determine the potential union between clusters including singletons. However, unlike the other compared clustering algorithms *Click* do not assume that the number of clusters is known and do not treat it as an input. In order to obtain a 'fair' comparison between solutions with the same number of clusters, we indirectly controlled this number by properly selecting the threshold for the average required homogeneity. In particular, we checked threshold values between 0.05 up to 1 in steps of 0.05 and selected the best obtained clustering solutions with 5, 6 and 7 clusters. Other parameters were defined by the Expander default values.

Note that another difference between *Click* and the other algorithms is the treatment of non-clustered singletons, which are allowed by *Click* without

affecting the obtained homogeneity and separation scores. The fact that not all the gene-expression profiles should be clustered provides a fundamental advantage for *Click*, in particular with respect to outliers and measuring errors that are commonly found in biological experiments.

2.4. ***Sequential Information Bottleneck (sIB)***

The sIB algorithms (sequential Information Bottleneck) was proposed by Slonim et al. (2002). The algorithm is based on the information bottleneck (IB) method which was originally suggested in Tishby et al. (1999) as a new information-theoretic approach for data analysis. Given the joint distribution p(*X*;*Y*), the IB method enables to produce a compact representation of *X*, which preserves as much information as possible about the relevant variable *Y*. The mutual information, I(*X*;*Y*), between the random variables *X* and *Y* is the natural statistical measure of the information that variable *X* contains about variable *Y* and vice versa. Denote by *T* the compressed representation of *X*. The compactness of the representation is now determined by I(*T*;*X*), while the quality of the clusters, *T*, is measured by the fraction of the information they capture about *Y*, namely, I(*T*;*Y*). The later is upper bounded by the compactness of the original system, I(*T*;*X*).

The sIB algorithm heuristically tries to maximize the following function:

$$I(T;Y)-\frac{1}{\beta}I(T;X),$$

where β is a trade-off parameter which controls the quality and the compactness of the solution. We used a "hard" clustering version of the sIB algorithm (Slonim, 2002, Slonim, 2005). In "hard" clustering each value of *X* is assigned to exactly one cluster in *T*. In this case, I(T;X) = H(T), where H(T) denotes the entropy of T. Thus, effectively, the sIB aims at maximizing

$$I(T;Y)-\frac{1}{\beta}H(T).$$

The sIB algorithm partitions the data into exactly K clusters. It starts from an initial random partition *T* of *X*. At each step, one element is drawn out of its current cluster and is ascribed to the cluster which maximize $\{ I(T;Y)-\frac{1}{\beta}H(T) \}$.

In order to avoid too slow convergence, Slonim et al. (2002) defines two "convergence" parameters denoted by *maxL* and ε. Specifically, the algorithm is converged if it already performed *maxL* loops over *X*, or if in the last loop there

were less than $\lfloor \varepsilon \cdot |X| \rfloor$ assignment changes, where $0 < \varepsilon < 1$. The preliminary joint distribution used as input for the sIB algorithm was create through markovian relaxation as described in Tishby and Slonim (2000). This method uses the pairwise Pearson correlation distance matrix as an input and transforms it to a Markovian Transition Matrix by an exponential transformation. The sIB was implemented by *IBA_1.0* (Slonim, 2005), available at *http://www.princeton. edu/~nslonim/*.

In the following Tables we detail the input parameters and the produced parameters of each of the four algorithms.

The Click algorithm:

Hm threshold	# clusters	singletons	*Hm* 6 Bins	*Sp* 6 Bins	*Hm* 8 Bins	*Sp* 8 Bins	*Hm* 10 Bins	*Sp* 10 Bins
default	7	21	0.24169	0.29078	0.45657	0.51845	0.70291	0.75338
0.10	3	17	0.23127	0.35433	0.44759	0.55706	0.69155	0.78177
0.15	3	20	0.22889	0.32317	0.44712	0.53366	0.68909	0.77702
0.20	3	25	0.23687	0.39603	0.44828	0.54716	0.69596	0.78922
0.25	3	23	0.24037	0.31832	0.44920	0.52954	0.69321	0.71409
0.30	3	24	0.23587	0.34114	0.44607	0.49356	0.69279	0.69824
0.35	3	23	0.23471	0.37196	0.45284	0.52084	0.69507	0.72077
0.40	6	58	0.24831	0.28445	0.46158	0.52460	0.70525	0.77752
0.45	6	55	0.24892	0.29085	0.46542	0.53051	0.70554	0.76502
0.50	6	53	0.25031	0.27937	0.46589	0.53163	0.70656	0.75976
0.55	6	53	0.25121	0.26474	0.46557	0.51919	0.70871	0.75935
0.60	6	54	0.24787	0.28240	0.46280	0.51407	0.70997	0.75994
0.65	6	55	0.24668	0.27333	0.46601	0.51978	0.70728	0.74247
0.70	5	127	0.25125	0.30329	0.46659	0.54561	0.70591	0.73816
0.75	5	155	0.24928	0.29091	0.46517	0.55445	0.70276	0.75287
0.80	5	185	0.24093	0.29512	0.45625	0.53266	0.70411	0.74684
0.85	5	187	0.24151	0.29268	0.45759	0.54571	0.70568	0.74785
0.90	5	183	0.24561	0.28938	0.45650	0.53926	0.70301	0.75075
0.95	5	189	0.24350	0.32629	0.45922	0.53036	0.70227	0.73273
1.00	8	18	0.25530	0.28749	0.46947	0.53891	0.70951	0.74650

The sIB algorithm:

# clusters	*z*	*f*	$\log_2 S$	β	*Hm* 6 Bins	*Sp* 6 Bins	*Hm* 8 Bins	*Sp* 8 Bins	*Hm* 10 Bins	*Sp* 10 Bins
5	80	30	25	280	0.25838	0.24531	0.46979	0.46767	0.71266	0.69713
5	80	30	25	290	0.26150	0.28997	0.47785	0.48980	0.72031	0.76183
5	80	30	25	310	0.25637	0.27403	0.47384	0.50786	0.70901	0.69405
5	80	30	25	320	0.26458	0.23024	0.46417	0.50081	0.70638	0.73061
5	80	30	25	330	0.26159	0.25949	0.46895	0.45765	0.70863	0.67143
5	80	30	25	340	0.26122	0.35772	0.47586	0.57770	0.71282	0.78786
5	80	30	25	360	0.25872	0.28036	0.46614	0.50428	0.70634	0.70674
5	80	30	25	390	0.25634	0.30731	0.47292	0.51264	0.70880	0.70086
5	80	30	25	480	0.25994	0.29151	0.47194	0.51578	0.71246	0.73323
5	80	30	25	490	0.26565	0.28153	0.48097	0.48550	0.71547	0.71795
6	80	30	25	50	0.26823	0.27291	0.47991	0.50787	0.71958	0.75257
6	80	30	25	60	0.27304	0.27759	0.48068	0.53927	0.71823	0.73699
6	80	30	25	80	0.27494	0.29827	0.48252	0.53408	0.72145	0.75784
6	80	30	25	130	0.27840	0.26334	0.48968	0.51403	0.72199	0.75015
6	80	30	25	280	0.27695	0.29977	0.48325	0.50691	0.72305	0.74938
6	80	30	25	320	0.27373	0.28286	0.48503	0.47686	0.72046	0.73753
6	80	30	25	340	0.27222	0.26279	0.47744	0.48046	0.71514	0.70604
6	80	30	25	440	0.27189	0.28581	0.47994	0.48792	0.72410	0.74051
6	80	30	25	460	0.27009	0.27593	0.48362	0.54086	0.71844	0.74876
6	80	30	25	520	0.26965	0.29722	0.47819	0.50686	0.72231	0.72491
7	80	30	25	100	0.27995	0.32438	0.48596	0.54331	0.72631	0.74912
7	80	30	25	130	0.26550	0.26684	0.47864	0.50481	0.71733	0.73907
7	80	30	25	140	0.27549	0.29405	0.48442	0.52569	0.72237	0.72375
7	80	30	25	190	0.26615	0.28632	0.47899	0.50789	0.71714	0.72377
7	80	30	25	350	0.27345	0.31372	0.48215	0.51081	0.71736	0.74974
7	80	30	25	560	0.26881	0.30740	0.48093	0.51510	0.71928	0.73731
7	80	30	25	580	0.26763	0.29057	0.47851	0.55394	0.71701	0.72640
7	80	30	25	600	0.26680	0.28215	0.48262	0.50047	0.71799	0.72720
7	80	50	25	120	0.27422	0.29716	0.48818	0.50744	0.72171	0.73240
7	80	50	25	530	0.27421	0.27639	0.47862	0.49504	0.71987	0.73022

The *K*-means algorithm:

# Clusters	# Initiations	*Hm* 6 Bins	*Sp* 6 Bins	*Hm* 8 Bins	*Sp* 8 Bins	*Hm* 10 Bins	*Sp* 10 Bins
5	500	0.24933	0.28221	0.46201	0.48764	0.71008	0.73147
6	500	0.24580	0.26820	0.45929	0.50418	0.70304	0.76107
7	500	0.25553	0.28947	0.47041	0.49857	0.71056	0.73827

The SOM algorithm:

# Clusters	Net	α_{init}	α_{fin}	σ_{init}	σ_{fin}	*Hm* 6 Bins	*Sp* 6 Bins	*Hm* 8 Bins	*Sp* 8 Bins	*Hm* 10 Bins	*Sp* 10 Bins
5	1X5	0.05	0.005	5	0.5	0.24629	0.30367	0.45991	0.4881	0.70321	0.76801
5	1X5	0.10	0.005	5	0.5	0.24581	0.31332	0.45967	0.48631	0.70233	0.76283
5	1X5	0.15	0.005	5	0.5	0.24581	0.31332	0.45967	0.48631	0.70233	0.76283
5	1X5	0.20	0.005	5	0.5	0.24601	0.28475	0.46076	0.51626	0.70501	0.77846
5	1X5	0.25	0.005	5	0.5	0.24581	0.31332	0.45967	0.48631	0.70233	0.76283
5	1X5	0.30	0.005	5	0.5	0.24408	0.28400	0.46041	0.52526	0.70432	0.76543
5	1X5	0.05	0.005	10	0.5	0.2507	0.30177	0.46417	0.50476	0.71208	0.7521
5	1X5	0.10	0.005	10	0.5	0.24581	0.26544	0.46135	0.51446	0.70445	0.77047
5	1X5	0.15	0.005	10	0.5	0.2507	0.30177	0.46417	0.50476	0.71208	0.7521
5	1X5	0.20	0.005	10	0.5	0.24581	0.26544	0.46135	0.51446	0.70445	0.77047
5	1X5	0.25	0.005	10	0.5	0.24581	0.26544	0.46135	0.51446	0.70445	0.77047
5	1X5	0.30	0.005	10	0.5	0.2549	0.33739	0.46191	0.51684	0.70633	0.77145
6	1X6	0.05	0.005	5	0.5	0.25845	0.29951	0.46872	0.51498	0.71027	0.76034
6	1X6	0.10	0.005	5	0.5	0.2587	0.29851	0.46808	0.51528	0.7096	0.75574
6	1X6	0.15	0.005	5	0.5	0.25576	0.33977	0.46505	0.50699	0.70326	0.77374
6	1X6	0.20	0.005	5	0.5	0.2587	0.29851	0.46808	0.51528	0.7096	0.75574
6	1X6	0.25	0.005	5	0.5	0.2587	0.29851	0.46808	0.51528	0.7096	0.75574
6	1X6	0.30	0.005	5	0.5	0.2587	0.29851	0.46808	0.51528	0.7096	0.75574
6	1X6	0.05	0.005	10	0.5	0.26563	0.32822	0.47236	0.53596	0.71415	0.79173
6	1X6	0.10	0.005	10	0.5	0.26141	0.30427	0.46863	0.53769	0.70986	0.77031
6	1X6	0.15	0.005	10	0.5	0.26216	0.31036	0.47101	0.51643	0.71207	0.75382
6	1X6	0.20	0.005	10	0.5	0.2621	0.31372	0.46919	0.53056	0.71183	0.7653
6	1X6	0.25	0.005	10	0.5	0.26356	0.30808	0.47097	0.51363	0.71404	0.77028
6	1X6	0.30	0.005	10	0.5	0.26356	0.30808	0.47097	0.51363	0.71404	0.77028
6	2X3	0.05	0.005	5	0.5	0.26563	0.32822	0.47236	0.53596	0.71415	0.79173
6	2X3	0.10	0.005	5	0.5	0.26563	0.32822	0.47236	0.53596	0.71415	0.79173
6	2X3	0.15	0.005	5	0.5	0.26563	0.32822	0.47236	0.53596	0.71415	0.79173
6	2X3	0.20	0.005	5	0.5	0.26563	0.32822	0.47236	0.53596	0.71415	0.79173
6	2X3	0.25	0.005	5	0.5	0.26563	0.32822	0.47236	0.53596	0.71415	0.79173
6	2X3	0.30	0.005	5	0.5	0.24609	0.3082	0.45775	0.50997	0.7005	0.75304
6	2X3	0.05	0.005	10	0.5	0.26552	0.32823	0.47222	0.5366	0.7143	0.80128
6	2X3	0.10	0.005	10	0.5	0.26563	0.32822	0.47236	0.53596	0.71415	0.79173
6	2X3	0.15	0.005	10	0.5	0.26563	0.32822	0.47236	0.53596	0.71415	0.79173
6	2X3	0.20	0.005	10	0.5	0.26563	0.32822	0.47236	0.53596	0.71415	0.79173
6	2X3	0.25	0.005	10	0.5	0.26563	0.32822	0.47236	0.53596	0.71415	0.79173
6	2X3	0.30	0.005	10	0.5	0.26563	0.32822	0.47236	0.53596	0.71415	0.79173
7	1X7	0.05	0.005	5	0.5	0.26331	0.31801	0.47573	0.54253	0.71188	0.74504
7	1X7	0.10	0.005	5	0.5	0.26322	0.31539	0.47345	0.54016	0.71574	0.74651
7	1X7	0.15	0.005	5	0.5	0.2586	0.32094	0.46662	0.51362	0.70931	0.75217
7	1X7	0.20	0.005	5	0.5	0.26322	0.31539	0.47345	0.54016	0.71574	0.74651
7	1X7	0.25	0.005	5	0.5	0.25365	0.30196	0.46382	0.50283	0.70445	0.74399
7	1X7	0.30	0.005	5	0.5	0.25365	0.30196	0.46382	0.50283	0.70445	0.74399
7	1X7	0.05	0.005	10	0.5	0.26692	0.34447	0.47339	0.52321	0.70908	0.75413
7	1X7	0.10	0.005	10	0.5	0.26675	0.31816	0.47436	0.54741	0.71005	0.7469
7	1X7	0.15	0.005	10	0.5	0.26615	0.31475	0.47357	0.54501	0.7124	0.75965
7	1X7	0.20	0.005	10	0.5	0.26615	0.31475	0.47357	0.54501	0.7124	0.75965
7	1X7	0.25	0.005	10	0.5	0.26675	0.31816	0.47436	0.54741	0.71005	0.7469
7	1X7	0.30	0.005	10	0.5	0.26322	0.3196	0.4752	0.5462	0.71015	0.73995

3. Comparison figures for clustering solutions

In this section we compare the results for 5, 6 and 7 clusters in Figures 3(a), 3(b) and 3(c) respectively. The axes indicate the homogeneity and separation scores. A clustering solution is considered better as the homogeneity score increases while the separation score decreases. The figures present the best clustering. Thus, for each algorithm, the figures present only those solutions that are not dominated by any other solution of the same algorithm. Whenever there are more than one un-dominated clustering solutions per algorithm, they form an efficiency frontier on the MI-based homogeneity-separation plane. This manner of evaluation of clustering solutions is accepted when the "true" clustering is unknown (e.g., Shamir and Sharan, 2002).

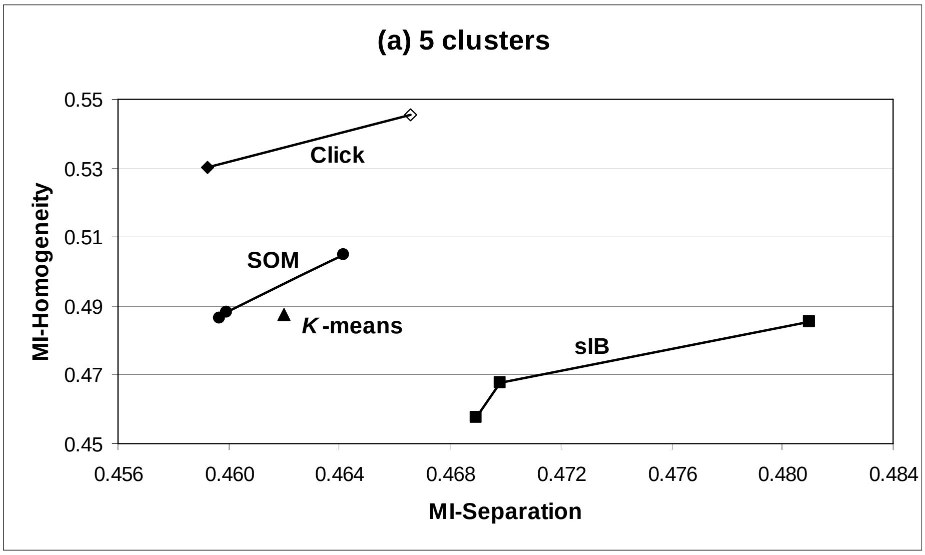

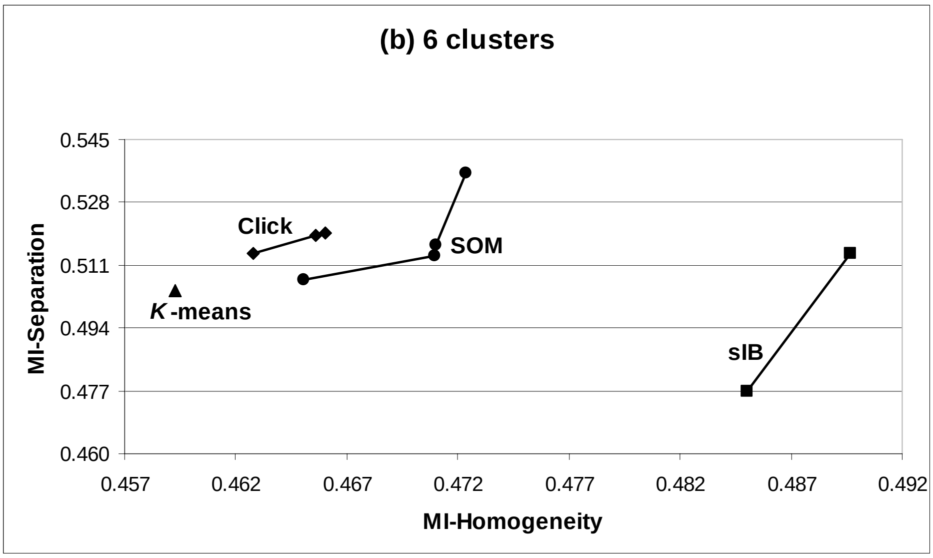

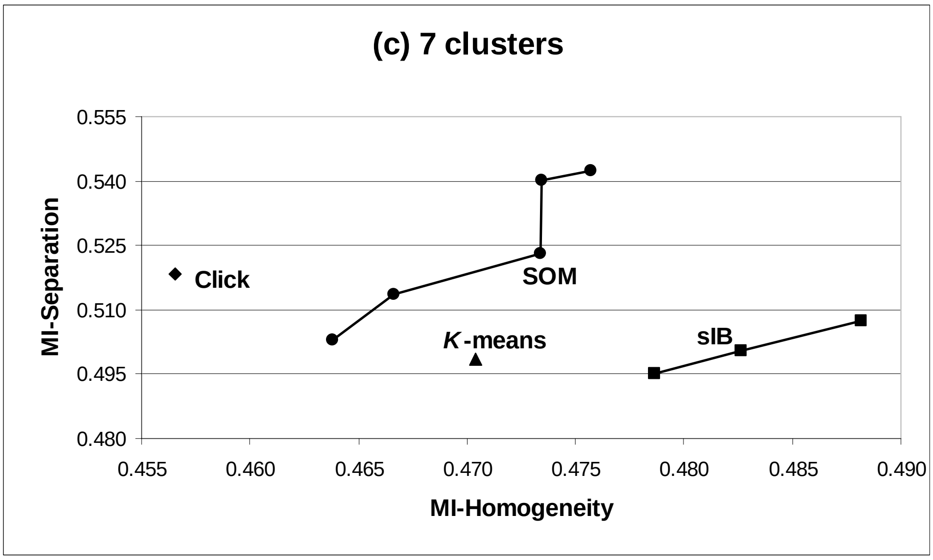

Fig. 3. Efficiency frontiers for solutions with 5, 6 and 7 clusters, as obtained by the *K-means*, the *SOM*, the *sIB* and the *Click* algorithms.

References

Daub,C.O., Steuer,R., Selbig,J. and Kloska,S. (2004) Estimating mutual information using B-spline functions – an improved similarity measure for analyzing gene expression data. *BMC Bioinformatics* 2004, 5:118.

Everitt,B. (1993) Cluster analysis. Edward Arnold, London.

Hansen,P. and Jaumard,B. (1997) Cluster analysis and mathematical programming. *Mathematical Programming*, 79,191-215.

Kohonen,T. (1997) Self-Organizing Maps. *Springer*, Berlin.

Law,A.M. and Kelton,W.D. (1991) *Simulation modeling & analysis*. McGraw-Hill Co., New-York.

MacQueen,J. (1965) Some methods for classification and analysis of multivariate observations. *In Proc. of the 5th Berkeley Symposium on Mathematical Statistics* and Probability, 281-297.

Mirkin,B. (1996) *Mathematical classification and clustering*. Kluwer Academic Publishers, Boston.

Shamir,R. and Sharan,R., (2002) Algorithmic approaches to clustering gene expression data. In T. Jiang, et al. eds., *Current Topics in Computational Biology*, MIT Press.

Sharan,R. and Shamir,R. (2000) CLICK: a clustering algorithm with applications to gene expression analysis. *Proc. 8th International Conference on Intelligent Systems for Molecular Biology*, 307-316.

Sharan,R., Maron-Katz,A. and Shamir,R. (2003) CLICK and EXPANDER: a system for clustering and visualizing gene expression data. *Bioinformatics*, **19**, 1787-1799.

Slonim N. (2005). Software: "IBA_1.0: Matlab Code for Informa-tion Bottleneck Clustering Algorithms", available at http://www.princeton.edu/~nslonim/.

Slonim,N. (2002). The information bottleneck: Theory and appli-cations. Ph.D. Thesis, available through http://www.cs.huji.ac.il/~tishby./

Slonim,N., Friedman,N. and Tishby,N. (2002) Unsupervised document classification using sequential information maximization. *In the 25th Annual International ACM SIGIR Conference on Research and Development in Information Retr*ieval (SIGIR).

Steuer,R., Kurths,J., Daub,C.O., Weise,J. and Selbig,J. (2002) The mutual information: Detecting and evaluating dependencies between variables. *Bioinformatics*, 18, 231–240.

Sturges,H.A. (1926) The choice of a class interval. *Journal of the American Statistical Association*, 21, 65-66.

Tamayo,P., Slonim,D., Mesirov,J., Zhu,Q., et al. (1999) Interpreting patterns of gene expression with self-organizing maps: Methods and application to hematopoietic differentiation. *PNAS*, **96**, 2907-2912.

Tishby,N. and Slonim,N. (2000) Data clustering by markovian relaxation and the information bottleneck method. *In advances in Neural Information Processing Systems (NIPS- 13)*, available through http://www.cs.huji.ac.il/~tishby/

Tishby,N., Pereira,F. and Bialek,W. (1999) The information bottleneck method. *In Proc. 37th Allerton Conference on Communication and Computation*.
